# Disparities in Reporting a History of Cardiovascular Disease Among Adults With Limited English Proficiency and Angina

**DOI:** 10.1001/jamanetworkopen.2021.38780

**Published:** 2021-12-14

**Authors:** Brandon M. Herbert, Amber E. Johnson, Michael K. Paasche-Orlow, Maria M. Brooks, Jared W. Magnani

**Affiliations:** 1 Department of Epidemiology, Graduate School of Public Health, University of Pittsburgh, Pittsburgh, Pennsylvania; 2 School of Medicine, University of Pittsburgh, Pittsburgh, Pennsylvania; 3 Department of Medicine, University of Pittsburgh, Pittsburgh, Pennsylvania; 4 Boston University School of Medicine, Boston, Massachusetts

## Abstract

**Importance:**

Individuals with limited English proficiency (LEP) may be unaware of underlying cardiovascular disease (CVD) owing to a lack of diagnostic testing or poor communication with health care practitioners.

**Objective:**

To evaluate whether participants with anginal symptoms and LEP would be less likely to report a history of CVD compared with those without LEP.

**Design, Study, and Participants:**

This population-based cross-sectional study combined data from 5 National Health and Nutrition Examination Survey (NHANES) cycles conducted from 2007 to 2016. Each cycle includes an interview that collects demographic, dietary, and health-related data as well as a medical examination component in which physiological measurements are taken. All NHANES participants aged 40 years or older who took the Rose questionnaire were included. Data were analyzed from September 2020 to April 2021.

**Exposures:**

LEP was defined as a participant receiving the survey in a non-English language or by interpreter.

**Main Outcomes and Measures:**

The 7-item Rose questionnaire assessed the presence of anginal symptoms. Self-reported CVD was defined as history of heart failure, coronary heart disease, angina pectoris, or myocardial infarction. The association between LEP status and self-reported CVD among those with anginal symptoms was determined in multivariable-adjusted models. All analyses were weighted per NHANES analytic protocols.

**Results:**

Among 19 320 participants (mean [SD] age, 57.8 [11.8] years; 9344 [47.2%] male; 4145 [10.6%] Black; 2743 [6.3%] Mexican American; 2111 [4.6%] other Hispanic; 8386 [71.6%] White; and 1935 [6.9%] other race), 583 (3.0%) reported anginal symptoms. Of these, most were non-LEP (484 [96.1%]), women (344 [62.1%]), White (251 [66.8%]), and did not report having CVD (347 [62.8%]). Among those with angina, 73 of 99 respondents with LEP (79.0%) reported not having a history of CVD, compared with 274 of 484 without LEP (61.4%; *P* = .002). Participants with LEP had 2.8-fold higher odds of not reporting a history of CVD compared with participants without LEP (odds ratio, 2.77; 95% CI, 1.38-5.55; *P* = .005).

**Conclusions and Relevance:**

Among NHANES participants reporting anginal symptoms, participants with LEP were more likely not to report having CVD. This discrepancy may be because of higher rates of undiagnosed CVD or lower awareness of such diagnoses among individuals with LEP. Our findings highlight the relevance of communication strategies for individuals with LEP to provide effective intervention and treatment for CVD prevention.

## Key Points

**Question:**

Is limited English proficiency (LEP) associated with not reporting a history of cardiovascular disease (CVD) among those with anginal symptoms?

**Findings:**

In this cross-sectional analysis of 583 participants in the National Health and Nutrition Examination Survey who had anginal symptoms, participants with LEP had significantly greater odds of not reporting a history of CVD compared with those without LEP in multivariable-adjusted models.

**Meaning:**

These findings highlight the importance of reducing disparities in treatment and CVD prevention for individuals with LEP.

## Teaser

This cross-sectional study evaluates whether participants with anginal symptoms and limited English proficiency would be less likely to report a history of cardiovascular disease compared with those without limited English proficiency.

## Introduction

The overwhelming majority of US residents speak English, but approximately 9% of the US population is classified as having limited English proficiency (LEP).^1^ People with LEP experience significant health care barriers.^2,3,4^ Language barriers have been associated with increased hospital length of stay,^5,6^ hospital readmissions,^7,8^ and limited understanding of appointment type and postdischarge medication use.^9^ In the international setting, the consequences of language barriers have been documented across countries in Europe, Africa, and the Middle East, with findings similar to those detected in the United States.^10^

Importantly, LEP and other language barriers are fundamentally detrimental to health literacy, further associated with poor health, adverse outcomes, and limited communication between the patient and health care practitioner.^8,11,12^ In a population-based sample, respondents with both LEP and low health literacy reported the highest prevalence of poor health when compared with those described as being non-LEP or having adequate health literacy.^11^ Even with the use of interpreters, individuals with LEP have reported difficulty understanding interpreters and feeling that interpreters omitted significant information during interactions.^13^ A recent systematic review investigating hospital-based interventions to improve communication, quality of care, and health outcomes for individuals with LEP concluded that more research is needed to address the gaps in language services and communication quality provided to individuals with LEP.^14^

Other factors also contribute to limited health care access and poor health outcomes for individuals with LEP. Individuals with LEP have reported racial and linguistic discrimination in the health care setting and avoid care as a result of these experiences.^2^ Patients without legal immigration status have hesitated to receive emergency medical attention. Additionally, health care practitioners may provide interventions and care that may be culturally insensitive or inappropriate.^2^ Suggestions to change certain diet or lifestyle behaviors may degrade the patient-practitioner relationship. Being sensitive to a patient’s background, culture, and experience is critical to providing high quality patient-centered care and reducing poor health outcomes.^12^

Addressing gaps in research for individuals with LEP presents several challenges given that few recurring national health surveys actively enroll LEP participants. The National Health and Nutrition Examination Survey (NHANES) is one of the few national research programs that facilitates studying individuals with LEP in the US population. NHANES is administered primarily in English and Spanish and, if needed, provides interpreters to assist its completion in other languages.^15^

The association between LEP and cardiovascular disease (CVD) outcomes has had limited investigation. A prior study used NHANES data to document the association of uncontrolled hypertension in individuals with LEP compared with those without LEP.^16^ NHANES data provide additional opportunities to assess how LEP may be associated with disparities between symptoms of CVD and self-reported history. Angina is widely identified as a symptom of heart disease and is critical in the recognition and prevention of CVD.^17,18,19^ For individuals with LEP and a history of CVD, recognition and accurate reporting of their history may affect evaluation and secondary prevention. We therefore assessed the association between LEP and self-reported history of CVD among NHANES participants with anginal symptoms. We hypothesized that among NHANES participants with angina, people with LEP would be more likely not to report a history of CVD than those without LEP.

## Methods

### Study Sample and Definitions

NHANES is conducted by the National Center for Health Statistics (NCHS) of the Centers of Disease Control and Prevention. NHANES consists of samples designed to assess the health and nutrition status of a subpopulation of the United States that is weighted and stratified to be generalizable to the entire noninstitutionalized population.^20^ The survey is conducted in 3 phases: screening, an in-home interview, and a mobile examination center examination.^15^ Further publications describing the survey methods and operations are available on the NHANES website.^20^ Informed consent was obtained from all participants, and the NCHS Research Ethics Review Board approved NHANES to conduct the surveys and examinations. This study followed the Strengthening the Reporting of Observational Studies in Epidemiology (STROBE) guidelines for cross-sectional studies.

We included publicly available data from five 2-year NHANES cycles spanning 2007 to 2016. A complete list of NHANES files used for analyses are detailed in the eAppendix in the Supplement. LEP was defined as taking the interview instrument in a language other than English or the use of an interpreter during the interview. The NHANES database includes a notation of whether interpretation services were used for each participant.^21^ Self-reported CVD was defined by a participant indicating a history of heart failure, coronary heart disease, angina and/or angina pectoris, or myocardial infarction.

Overall, 19 320 survey participants aged 40 years or older received the cardiovascular health questionnaire and were included in this analysis. The cardiovascular health questionnaire includes the 7-item Rose questionnaire, an inexpensive and reliable instrument that has undergone extensive validation to identify the presence of symptoms consistent with angina and ischemic heart pain.^22^ The questionnaire has high specificity (97%) and moderate sensitivity (approximately 83%) to detect angina pectoris, myocardial infarction, and intermittent claudication.^17,22^ Additionally, the questionnaire has demonstrated value in detecting coronary heart disease and myocardial infarction in diverse populations.^23,24,25,26,27^ Specifically, the Rose questionnaire has been studied in Spanish-speaking populations and was found to be a reliable tool.^27^ Definitions for grade of angina are defined in the eAppendix in the Supplement.

Race and ethnicity was classified into Mexican American, non-Hispanic Black, non-Hispanic White, other Hispanic, and other race (ie, American Indian or Alaskan Native; Native Hawaiian or Pacific Islander; multiple races or ethnicities; or unknown). Dichotomous ethnicity (Hispanic or non-Hispanic) was created for regression analyses. Body mass index (BMI; calculated as weight in kilograms divided by height in meters squared) and mean blood pressure over 3 measurements, both obtained at the NHANES standardized examination, were analyzed as continuous variables. Diabetes was defined through self-report, and those with borderline diabetes were classified as not having the condition. We also included health insurance (yes or no), number of health care visits over the past 12 months (0, 1-3, 4-9, and ≥10), ratio of family income to poverty (poverty-income ratio), and educational attainment (≤high school or >high school equivalent). The poverty-income ratio was calculated by NHANES and uses the Department of Health and Human Services poverty guidelines specific to family size and geographic location.^21^

### Statistical Analysis

Categorical variables are presented as crude frequencies and weighted proportions, and continuous variables are expressed as means with the associated SD or 95% CI. We examined the characteristics of the entire NHANES cohort that completed the cardiovascular health questionnaire and the subset that screened positive for angina on the Rose questionnaire, stratified by LEP status. χ^2^ tests and *t* tests were performed to assess differences in the characteristics between the non-LEP and LEP groups for categorical and continuous variables, respectively. Multicollinearity between LEP and race and ethnicity were assessed using χ^2^ tests. The proportion of participants self-reporting CVD who screened positive on the Rose questionnaire for each survey year was plotted by LEP status, and an unadjusted linear regression model was used to estimate the linear relationships with 95% CIs using Prism version 8.4.3 (GraphPad).

We used multivariable logistic regression to associate LEP status with whether participants did not report a history of CVD. Missing data were incorporated into each analysis and considered not missing completely at random.^28^ This was performed by adding the NOMCAR option to all models in SAS version 9.4 (SAS Institute). Four models were constructed with the following independent variables: model 1 (LEP; age [per 10 years], sex, race and ethnicity), model 2 (model 1 as well as BMI [continuous], mean systolic blood pressure [per 5 mm Hg], mean diastolic blood pressure [per 5 mm Hg], and diabetes), and model 3 (model 2 as well as health insurance coverage, health care utilization, poverty-income ratio, and educational attainment). Model 4 was constructed using a backward stepwise selection method in which a fixed threshold of *P* < .20 was used. As sex also exhibited significant associations in these analyses, a fifth model including an interaction term of LEP and sex with all covariates specified in model 4 was examined. Given the limited number of individuals of races other than White with LEP, we opted not to include race in our multivariable adjustment. To adjust for the combination of 5 NHANES cycles, we divided each participant’s weight by a factor of 5, per NHANES analytics protocol.^29^

Sensitivity analyses were conducted to (1) determine the association between race and ethnicity and self-reported CVD without LEP in regression models; (2) model self-reported myocardial infarction, rather than all CVD conditions, given that the Rose questionnaire has particularly high sensitivity for myocardial infarction; and (3) evaluate the agreement of diabetes status with fasting plasma glucose levels in a subset of the sample to evaluate the possibility of differential reporting of diabetes status across LEP.

For all analyses, a 2-tailed *P* ≤ .05 was considered significant. All analyses used the complex survey procedures in SAS version 9.4 (SAS Institute) in which sample weights and variances were incorporated to produce nationally representative estimates and account for the survey design of NHANES.^29,30^

## Results

Table 1 presents the demographic profile for the 19 320 participants who completed the Rose questionnaire, including 9976 (52.8%) women, 4145 (10.6%) Black individuals; 2743 (6.3%) Mexican American individuals; 2111 (4.6%) other Hispanic individuals; 8386 (71.6%) White individuals; and 1935 (6.9%) individuals who identified as other race. The mean (SD) age was 57.7 (11.8) years. In total, 3369 participants (7.8%) were classified as having LEP. Overall, 583 (2.6%) screened positive on the Rose questionnaire, and this was similar for those with and without LEP (484 [2.6%] vs 99 [2.8%]; *P* = .87). Overall, 2385 (10.1%) reported a history of CVD, but participants with LEP were less likely to report CVD than participants without LEP (303 [7.3%] vs 2082 [10.4%]; *P* = .003).

**Table 1.** Characteristics of the NHANES Cohort Who Completed the Cardiovascular Health Questionnaire and Were Aged 40 Years and Older, 2007-2016, Stratified by English Proficiency

Characteristic	NHANES participants, No. (weighted %)			*P* value, weighted
	All (N = 19 320)	English proficient (n = 15 951)	Limited English proficiency (n = 3369)	
Age, mean (SD)	57.7 (11.8)	57.9 (11.8)	55.2 (11.6)	<.001
Male sex	9344 (47.2)	7764 (47.2)	1580 (47.1)	.92
Female sex	9976 (52.8)	8187 (52.8)	1789 (52.9)	
Race/ethnicity				
Mexican American	2743 (6.3)	1248 (3.2)	1495 (43.1)	<.001
Non-Hispanic Black	4145 (10.6)	4098 (11.4)	47 (1.41)	
Non-Hispanic White	8386 (71.6)	8314 (77.3)	72 (4.2)	
Other Hispanic	2111 (4.6)	915 (2.3)	1196 (32.0)	
Other race^a^	1935 (6.9)	1376 (5.8)	559 (19.3)	
BMI, mean (SD)	29.3 (6.5)	29.4 (6.6)	28.6 (5.3)	<.001
Blood pressure, mean (SD), mm Hg				
Systolic	126.1 (18.0)	126.0 (18.0)	127.1 (18.0)	.05
Diastolic	71.2 (12.4)	71.2 (12.4)	71.2 (11.8)	.95
Diabetes	3400 (13.9)	2733 (13.5)	667 (17.9)	<.001
Health insurance				
Yes	16 116 (87.3)	14 015 (89.7)	2101 (58.9)	<.001
No	3185 (12.7)	1923 (10.3)	1262 (41.1)	
Health care visits over past year				
0	2376 (11.7)	1653 (10.6)	723 (24.2)	<.001
1-3	8220 (44.9)	6810 (45.1)	1410 (43.4)	
4-9	5710 (29.2)	4910 (29.8)	800 (21.5)	
≥10	2995 (14.2)	2565 (14.5)	430 (10.9)	
Poverty-income ratio, mean (SD)	2.56 (1.63)	2.75 (1.64)	1.54 (1.16)	<.001
Education				
≤HS	9970 (40.8)	7227 (37.4)	2743 (80.6)	<.001
>HS	9323 (59.2)	8707 (62.6)	616 (19.4)	
Rose score				
Grade 0	18 737 (97.4)	15 467 (97.4)	3270 (97.2)	.87
Grade 1	300 (1.4)	247 (1.4)	53 (1.5)	
Grade 2	283 (1.2)	237 (1.2)	46 (1.2)	
Self-reported CVD				
Yes	2385 (10.1)	2082 (10.4)	303 (7.3)	.003
No	16 832 (89.9)	13 794 (89.6)	3038 (92.7)	
CVD diagnosis				
Congestive heart failure	937 (3.7)	820 (3.7)	117 (3.0)	.10
Coronary heart disease	1152 (5.1)	1002 (5.3)	150 (3.4)	<.001
Angina pectoris	692 (3.2)	601 (3.2)	91 (2.5)	.06
Myocardial infarction	1181 (5.0)	1046 (5.1)	135 (2.9)	<.001

Abbreviations: BMI, body mass index (calculated as weight in kilograms divided by height in meters squared); CVD, cardiovascular disease; HS, high school; NHANES, National Health and Nutrition Examination Survey.

^a^ Other race includes American Indian or Alaska Native; Native Hawaiian or Pacific Islander; multiple races or ethnicities; or unknown.

Of the 583 participants who screened positive on the Rose Questionnaire (Table 2), 99 (8.4%) were classified as having LEP. There was no statistical difference by anginal grade (ie, Rose score) (Table 2) across LEP and non-LEP groups. While rates of health insurance did vary by LEP status (LEP, 30 of 99 [35.3%]; no LEP, 60 of 484 [13.0%]; *P* < .001), the number of times individuals reported receiving care over the past year was similar. All 4 CVD diagnoses were reported in smaller proportions in the LEP group, but only myocardial infarction was found to differ significantly by LEP status. Among 583 people who reported angina, 347 (62.8%) did not report a CVD diagnosis. This discordance was higher for LEP vs non-LEP participants (73 of 99 [79.0%] vs 274 of 484 [61.4%]; *P* = .002).

**Table 2.** Characteristics for NHANES Participants Who Screened Positive on the Rose Questionnaire, Stratified by English Proficiency

Characteristic	NHANES participants, No. (weighted %)			*P* value, weighted
	All participants (n = 583)	English proficient (n = 484)	Limited English proficiency (n = 99)	
Age, mean (SD)	58.8 (11.7)	58.6 (11.8)	60.1 (11.4)	.37
Male sex	239 (37.9)	204 (38.5)	35 (31.4)	.23
Female sex	344 (62.1)	280 (61.5)	64 (68.6)	
Race/ethnicity				
Mexican American	72 (5.5)	31 (2.4)	41 (38.6)	<.001
Non-Hispanic Black	156 (15.4)	154 (16.5)	2 (2.4)^a^	
Non-Hispanic White	251 (66.8)	247 (72.0)	4 (9.5)^a^	
Other Hispanic	64 (5.2)	21 (2.0)	43 (40.2)	
Other race^b^	40 (7.2)	31 (7.0)	9 (9.3)^a^	
BMI, mean (SD)	31.3 (7.4)	31.3 (7.5)	31.2 (5.6)	.97
Blood pressure, mean (SD), mm Hg				
Systolic	126.7 (18.7)	126.3 (18.8)	131.8 (17.4)	.03
Diastolic	68.9 (13.1)	68.6 (13.2)	71.7 (11.8)	.04
Diabetes	163 (24.0)	135 (24.0)	28 (23.9)	.98
Health insurance				
Yes	492 (85.1)	424 (87.0)	68 (64.7)	<.001
No	90 (14.9)	60 (13.0)	30 (35.3)	
Health care visits over past year				
0	34 (6.0)	27 (5.8)	7 (7.7)^a^	.52
1-3	162 (28.1)	129 (27.6)	33 (34.7)	
4-9	223 (39.2)	194 (40.0)	29 (30.5)	
≥10	164 (26.6)	134 (26.6)	30 (27.1)	
Poverty-income ratio, mean (SD)	1.87 (1.37)	1.94 (1.42)	1.46 (1.01)	<.001
Education				
≤HS	378 (58.1)	294 (55.6)	84 (85.8)	<.001
>HS	205 (41.9)	190 (44.4)	15 (14.2)	
Rose score				
Grade 0	NA	NA	NA	.97
Grade 1	300 (54.9)	247 (54.9)	53 (54.7)	
Grade 2	283 (45.1)	237 (45.1)	46 (45.3)	
Self-reported CVD				
Yes	229 (37.2)	205 (38.6)	24 (21.0)	.002
No	347 (62.8)	274 (61.4)	73 (79.0)	
CVD diagnosis				
Congestive heart failure	96 (13.5)	86 (13.9)	10 (8.8)	.13
Coronary heart disease	119 (18.0)	103 (18.3)	16 (14.1)	.35
Angina pectoris	85 (14.0)	74 (14.3)	11 (9.9)	.31
Myocardial infarction	123 (21.3)	113 (22.4)	10 (9.1)	.003

Abbreviations: BMI, body mass index (calculated as weight in kilograms divided by height in meters squared); CVD, cardiovascular disease; HS, high school; NA, not applicable; NHANES, National Health and Nutrition Examination Survey.

^a^ Per National Center for Health Statistics guidelines, may be considered an unreliable estimate of the proportion (residual SE ≥30%).

^b^ Other race includes American Indian or Alaska Native; Native Hawaiian or Pacific Islander; multiple races or ethnicities; or unknown.

The Figure presents the proportion of participants with and without LEP who reported CVD among those who screened positive on the Rose questionnaire prospective cycles of NHANES administration from 2007 to 2016. There were differences in the rates of CVD (*F* = 7.8; P = .003) by LEP status, but the rate of change over NHANES cycle administrations was comparable.

**Figure.**
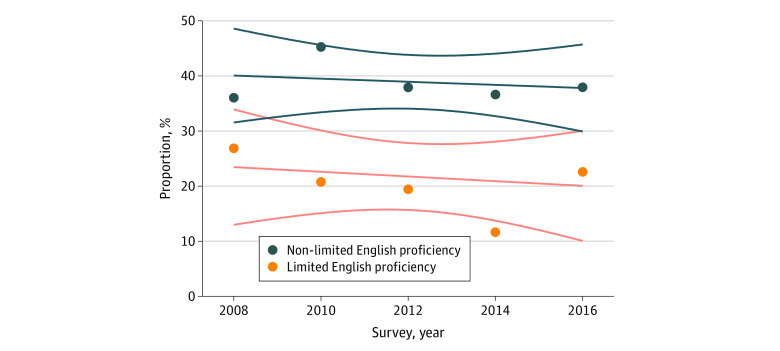
Trends in Self-reported Cardiovascular Disease Over Time Among 583 Participants Screening Positive on the Rose Questionnaire From Unadjusted Linear Regression Estimates Dots indicate proportions for each survey year; straight lines, linear regression estimate; curved lines, 95% CI bands.

Multivariable logistic regression results are shown in Table 3. In model 4, the final backward stepwise selection model, participants with LEP had 2.8 times higher odds of not reporting a previous diagnosis of CVD compared with participants without LEP (odds ratio, 2.77; 95% CI, 1.38-5.55; *P* = .005) after adjusting for age, sex, BMI, diastolic blood pressure, and diabetes status. Women were 2.6 times more likely not to report CVD than men (odds ratio, 2.63; 95% CI, 1.57-4.40). The C statistic for model 4 was 0.74, indicating good model fit. The fifth model, including an interaction term of the LEP and sex variables with model 4, did not show a significant interaction.

**Table 3.** Multivariable Logistic Regression Model of No Self-reported Diagnosis of Cardiovascular Disease Among 583 Participants Who Screened Positive on the Rose Questionnaire

Characteristic	Model 1		Model 2		Model 3		Model 4	
	OR (95% CI)	*P* value	OR (95% CI)	*P* value	OR (95% CI)	*P* value	OR (95% CI)	*P* value
Limited English proficiency	2.77 (1.32-5.81)	<.001	2.49 (1.10-5.66)	.03	2.32 (0.92-5.87)	.08	2.77 (1.38-5.55)	.005
Age (per 10 y)	0.54 (0.42-0.68)	<.001	0.57 (0.44-0.74)	<.001	0.55 (0.40-0.76)	<.001	0.56 (0.43-0.72)	<.001
Female	2.37 (1.42-3.95)	.001	2.65 (1.57-4.47)	<.001	2.50 (1.43-4.36)	.002	2.63 (1.57-4.40)	<.001
Hispanic	1.02 (0.52-1.98)	.96	0.86 (0.41-1.82)	.69	0.83 (0.40-1.73)	.62	NA	NA
BMI (per unit)	NA	NA	0.97 (0.94-1.00)	.06	0.96 (0.93-1.00)	.03	0.97 (0.94-1.00)	.06
Blood pressure (per 5 mm Hg)								
Mean systolic	NA	NA	0.99 (0.92-1.07)	.75	0.98 (0.91-1.06)	.66	NA	NA
Mean diastolic	NA	NA	1.09 (0.99-1.21)	.09	1.10 (0.99-1.22)	.08	1.09 (0.98-1.20)	.10
Diabetes	NA	NA	0.41 (0.24-0.72)	.002	0.43 (0.23-0.79)	.007	0.42 (0.24-0.73)	.003
No health insurance	NA	NA	NA	NA	0.69 (0.31-1.53)	.36		
Times health care received over past year (vs ≥10), No.								
0	NA	NA	NA	NA	1.57 (0.45-5.47)	.48	NA	NA
1-3	NA	NA	NA	NA	1.63 (0.72-3.66)	.24	NA	NA
4-9	NA	NA	NA	NA	0.89 (0.51-1.56)	.68	NA	NA
PIR (per point)	NA	NA	NA	NA	0.90 (0.72-1.13)	.36	NA	NA
HS education (vs ≤HS)	NA	NA	NA	NA	0.90 (0.48-1.67)	.73	NA	NA
C statistic	0.72		0.74		0.75		0.74	

Abbreviations: BMI, body mass index; HS, high school; NA, not applicable; OR, odds ratio; PIR, poverty-income ratio.

In sensitivity analyses, the detailed race and ethnicity variable was included as an independent variable (without LEP) using 3 similar models; no significant association was detected between race and ethnicity and reported CVD (eTable 1 in the Supplement). Participants with LEP had 4.6 times the odds of not reporting a previous diagnosis of myocardial infarction compared with participants without LEP (odds ratio, 4.58; 95% CI, 1.75-12.01) (eTable 2 in the Supplement). eTable 3 and eTable 4 in the Supplement detail the sensitivity analysis for diabetes; small but statistically significant differences were found in the agreement between self-reported diabetes status and fasting plasma glucose levels by LEP status.

## Discussion

In this nationally representative study incorporating 5 cycles of NHANES participants, we found that individuals with LEP were 2.8-fold more likely not to report having been diagnosed with CVD compared with those without LEP (odds ratio, 2.77; 95% CI, 1.38-5.55; *P* = .005). This association was independent of age, sex, BMI, diastolic blood pressure, and diabetes status. Our findings suggest that the rate of self-reported CVD is low across all NHANES participants reporting anginal symptoms. Indeed, two-thirds of those who screened positive on the Rose questionnaire did not report a CVD diagnosis. Furthermore, our findings exhibit that this discordance—reporting anginal symptoms without having been diagnosed with CVD—is much more likely for individuals with LEP.

In the full NHANES cohort, positive screening rates on the Rose questionnaire were similar across LEP status, suggesting a similar underlying prevalence of CVD. Similarly, grade of angina was very similar by LEP status, suggesting severity of symptoms and the likelihood of CVD (diagnosed or undiagnosed) was also similar. To be sure, with a proper evaluation, a person with positive Rose questionnaire screening could certainly be found to have or not have a cardiac diagnosis. However, it is important to note that the overall frequency of self-reported CVD (37.2%) was far lower than what would be anticipated given the sensitivity and specificity of the Rose questionnaire (eg, 81% and 97% for angina pectoris, respectively).^17^ This presents a major gap in care; many people have symptoms indicative of CVD but have not received a diagnosis. Accordingly, significant improvements are needed in screening and evaluation for CVD in the general US population.

Among the 64.2 million people who speak a language other than English, 25.7 million, or 8.5% of the entire population aged 5 years and older, are estimated to have LEP.^31^ Federal regulations, such as Title VI of the Civil Rights Act, are in place to protect individuals with LEP from discrimination and receipt of substandard health care. However, data indicate that some hospitals do not provide sufficient language services to patients with LEP and are thereby not compliant with federal law.^32^

Our findings are consistent with a previous study that analyzed English proficiency and postdischarge understanding of follow-up appointments and medication use.^9^ Our results are also congruent with data from the Hispanic Community Health Study/Study of Latinos, which identified that not speaking English as a primary language was strongly associated with suboptimal care, including not receiving guideline-directed therapies.^33^

The greater proportion of participants with LEP not reporting previous CVD diagnoses in our study is likely attributable to multiple factors. NHANES participants may have received a previous diagnosis from their health care practitioner but did not understand or were not able to recall this information during the study examination. Miscommunication of this kind could certainly be more common for individuals with LEP. Alternatively, participants with LEP may have never received a CVD diagnosis. Language discordance between practitioner and patient, inadequate use of interpreters, or other sociocultural barriers may lead to lower rates of appreciation of the presence of anginal symptoms and/or a higher rate of disregarding such symptoms or other disruptions to initiating or completing diagnostic activities. Another contributor to these findings may be that individuals with LEP may be overrepresented among those who remain outside of the health care system. Indeed, people with LEP in this study were much more likely to be uninsured compared with people with English proficiency (35.3% vs 13.0%); this could lead to barriers to diagnosis even though there did not appear to be a difference in the rate of seeing a health care practitioner in the past year.

Another pertinent finding is that women with a positive Rose questionnaire were 2.6 times more likely not to report CVD compared to men with a positive Rose questionnaire (odds ratio, 2.63; 95% CI, 1.57-4.40). This finding suggests that diagnostic screening and appropriate evaluations for CVD may need to be increased in women. These data potentially suggest that practitioners may not appreciate or otherwise disregard symptoms of CVD by female vs male patients. This is consistent with prior reports of symptoms of angina in women but low rates of diagnosed CVD.^18,34^

Through the national community-based sampling of NHANES, we were able to determine associations generalizable to the entire population of individuals identified as having LEP in the United States and not only the segment of those with LEP in contact with the health care system. Our study identifies a disparity in cardiovascular health for people with LEP. Gaps in screening, diagnosis, communication regarding CVD, and access to services can all lead to poor outcomes. Health care institutions should identify systems-level barriers to care for patients who do not communicate in the dominant language. By incorporating patient-centered practices, such as screening for and assessing risk for CVD in an individual’s preferred language, more patients may benefit from the equitable provision of guideline-directed therapies. Further research is needed to understand the burden of CVD in the US population with LEP and to assess for disparities in evaluation, treatment, and understanding of these conditions.

### Limitations

We acknowledge that our study has some important limitations. NHANES uses self-reporting of diagnoses. As such, the diagnoses used here may be subject to different types of response bias and result in misclassification of our outcome variable. However, the ability to recall and understand diagnoses was a primary interest of this study. Verification and adjudication of self-reported diagnosis is not possible when using NHANES data. Second, the number of participants with LEP was relatively small, such that our analyses were underpowered to determine reliable estimates in certain tabulations where respondent counts were excessively low (eg, by race and LEP). Our definition for LEP may lead to misclassification, as participants may have chosen to take the interview in a language other than English or use an interpreter when they could have completed the survey in English. Additionally, it is critical to recognize the heterogeneity within the English proficiency groups that were categorized into 2 strict categories (LEP vs non-LEP). The LEP group includes NHANES participants with multiple languages, and we expect large cultural differences exist in this cohort that were not accounted for in our analyses. The Rose questionnaire is known to perform differently across populations, and its sensitivity and specificity may vary across both sex and spoken language in this study.^34,35^

## Conclusions

Our findings suggest that disparities in cardiovascular health are associated with language barriers. We found that in a nationally representative cohort, LEP was associated with greater odds of not reporting a history of CVD among individuals with anginal symptoms. These findings highlight the importance of effective communication, screening, and diagnostic evaluations about heart disease in individuals with and without LEP.

## Notes

Supplement. **eTable 1.** Multivariable Logistic Regression Model of No Self-reported Diagnosis of CVD by Race/Ethnicity **eTable 2.** Multivariable Logistic Regression Modelling the Odds of No Self-reported Diagnosis of Myocardial Infarction Among Participants Who Screened Positive on the Rose Questionnaire **eTable 3.** Self-reported Diabetes Status and Fasting Plasma Glucose Levels Among the 2015-2016 NHANES Fasting Subsample **eTable 4.** English Proficiency Status and Agreement Between Self-reported Diabetes and Fasting Plasma Glucose Levels
eAppendix.
