# Supplementary material for: Disparities in Reporting a History of Cardiovascular Disease Among Adults With Limited English Proficiency and Angina
Source: JAMA Netw Open. 2021 Dec 14;4(12):e2138780. doi: 10.1001/jamanetworkopen.2021.38780 (PMC8672228; doi:10.1001/jamanetworkopen.2021.38780)
Supplement: Supplement. — eTable 1. Multivariable Logistic Regression Model of No Self-reported Diagnosis of CVD by Race/Ethnicity eTable 2. Multivariable Logistic Regression Modelling the Odds of No Self-reported Diagnosis of Myocardial Infarction Among Participants Who Screened Positive on the Rose Questionnaire eTable 3. Self-reported Diabetes Status and Fasting Plasma Glucose Levels Among the 2015-2016 NHANES Fasting Subsample eTable 4. English Proficiency Status and Agreement Between Self-reported Diabetes and Fasting Plasma Glucose Levels eAppendix. [file jamanetwopen-e2138780-s001.pdf]

Supplemental Online Content

Herbert BM, Johnson AE, Paasche-Orlow MK, Brooks MM, Magnani JW. Disparities in reporting a history of cardiovascular disease among adults with limited English proficiency and angina. *JAMA Netw Open.* 2021;4(12):e2138780. doi:10.1001/jamanetworkopen.2021.38780

eTable 1. Multivariable Logistic Regression Model of No Self-reported Diagnosis of CVD by Race and Ethnicity

eTable 2. Multivariable Logistic Regression Modelling the Odds of No Self-reported Diagnosis of Myocardial Infarction Among Participants Who Screened Positive on the Rose Questionnaire

eTable 3. Self-reported Diabetes Status and Fasting Plasma Glucose Levels Among the 2015-2016 NHANES Fasting Subsample

eTable 4. English Proficiency Status and Agreement Between Self-reported Diabetes and Fasting Plasma Glucose Levels

eAppendix.

This supplemental material has been provided by the authors to give readers additional information about their work.

© 2021 Herbert BM et al. *JAMA Network Open.*

Table 1. Multivariable Logistic Regression Model of No Self-reported Diagnosis of CVD by Race and Ethnicity									
	Model 1			Model 2			Model 3		
	OR	95% CI	p	OR	95% CI	p	OR	95% CI	p
Race/ethnicity (ref=NH White)			0.06			0.08			0.11
Mexican American	1.19	0.54-2.61	0.66	1.45	0.63-3.31	0.37	1.26	0.55-2.88	0.58
Other Hispanic	2.17	0.96-4.91	0.06	2.15	0.90-5.14	0.08	2.57	0.92-7.22	0.07
NH Black	0.75	0.41-1.39	0.36	0.86	0.48-1.55	0.61	0.90	0.48-1.67	0.73
Other race	0.42	0.15-1.23	0.11	0.35	0.11-1.13	0.08	0.28	0.08-1.01	0.05
Age (per 10-year)	0.53	0.41-0.68	<0.001	0.57	0.43-0.74	<0.001	0.55	0.40-0.76	<0.001
Female	2.34	1.40-3.91	0.002	2.62	1.55-4.44	<0.001	2.44	1.39-4.28	0.002
BMI (per kg/m^2)				0.97	0.94-1.00	0.06	0.96	0.93-1.00	0.03
Blood Pressure (per 5 mmHg)									
Mean SBP				1.00	0.98-1.01	0.65	1.00	0.98-1.01	0.53
Mean DBP				1.02	1.00-1.04	0.04	1.02	1.00-1.04	0.04
Diabetes				0.40	0.23-0.71	0.002	0.42	0.23-0.79	0.008
No Health Insurance							0.84	0.40-1.77	0.65
Number of times receive care over past year (ref=10+)									0.24
Zero							1.31	0.35-4.91	0.69
1-3							1.70	0.81-3.57	0.16
4-9							0.81	0.48-1.39	0.45
PIR (per point)							0.89	0.71-1.11	0.31
HS Education (versus ≤HS)							0.86	0.45-1.62	0.63
c-statistic	0.70			0.73			0.74		
OR, indicates odds ratio; CI, confidence interval; LEP, limited English Proficiency; BMI, body mass index; SBP, systolic blood pressure; DBP, diastolic blood pressure; PIR, poverty-income ratio; HS, high school.									

© 2021 Herbert BM et al. *JAMA Network Open.*

eTable 2. Multivariable Logistic Regression Modelling the Odds of No Self-reported Diagnosis of Myocardial Infarction Among Participants Who Screened Positive on the Rose Questionnaire									
	Model 1			Model 2			Model 3		
	OR	95% CI	p	OR	95% CI	p	OR	95% CI	p
Limited English Proficiency	4.08	1.81-9.20	0.004	3.64	1.57-8.43	0.007	4.58	1.75-12.01	0.004
Age (per 10-years)	0.67	0.50-0.90	0.01	0.77	0.57-1.06	0.12	0.74	0.53-1.02	0.08
Female	3.63	2.06-6.42	<0.001	3.95	2.18-7.18	<0.001	3.76	2.10-6.74	<0.001
Hispanic	0.72	0.37-1.39	0.24	0.72	0.36-1.46	0.40	0.68	0.31-1.47	0.26
BMI (per kg/m2)				1.00	0.96-1.04	0.90	0.99	0.95-1.03	0.65
Blood Pressure (per 5 mmHg)									
Mean SBP				1.00	0.98-1.01	0.61	1.00	0.98-1.01	0.53
Mean DBP				1.03	1.00-1.05	0.03	1.02	1.00-1.05	0.04
Diabetes				0.47	0.24-0.92	0.03	0.49	0.24-0.98	0.04
No Health Insurance							0.54	0.23-1.29	0.16
Number of times receive care over past year (ref=10+)									0.54
Zero							2.12	0.45-9.95	0.34
1-3							1.61	0.62-4.23	0.33
4-9							1.57	0.82-3.04	0.17
PIR (per point)							0.86	0.67-1.11	0.28
HS Education (versus ≤HS)							0.91	0.47-1.75	0.82
c-statistic	0.71			0.72			0.71		
OR, indicates odds ratio; CI, confidence interval; LEP, limited English Proficiency; BMI, body mass index; SBP, systolic blood pressure; DBP, diastolic blood pressure; PIR, poverty-income ratio; HS, high school.									

© 2021 Herbert BM et al. *JAMA Network Open.*

Diabetes sensitivity analysis:

eTable 3. Self-reported Diabetes Status and Fasting Plasma Glucose Levels Among the 2015-2016 NHANES Fasting Subsample				
	Self-Reported Diabetes Status			
	English Proficient		Limited English Proficiency	
	Yes	No	Yes	No
Fasting Plasma Glucose (mmol/L), n (%)				
<5.7	55 (9.0)	3333 (76.3)	8 (6.4)	599 (64.8)
5.7≤FPG<6.5	142 (26.7)	1142 (21.9)	41 (24.3)	305 (30.9)
6.5≤	400 (64.3)	103 (1.75)	145 (69.2)	49 (4.3)

FPG, fasting plasma glucose; NHANES, National Health and Nutrition Examination Survey.

© 2021 Herbert BM et al. *JAMA Network Open.*

eTable 4. English Proficiency Status and Agreement Between Self-reported Diabetes and Fasting Plasma Glucose Levels			
	English Proficient	Limited English Proficiency	p
Agreement, n (%)			<0.01
Yes	4875 (95.0)	1049 (92.1)	
No	300 (5.0)	98 (7.9)	

Agreement defined as a fasting plasma glucose level of 6.5≤ mmol/L indicative of diabetes; p-value obtained through a chi-square test.

© 2021 Herbert BM et al. *JAMA Network Open.*

eAppendix.

NHANES Data Files

The following list names the NHANES data files used for this analysis. Each data file was downloaded for each of the five NHANES cycles that spans 2007-2016:

1. Demographic Variables and Sample Weights
2. Blood Pressure
3. Body Measures
4. Cardiovascular Health
5. Diabetes
6. Health Insurance
7. Hospital Utilization & Access to Care
8. Medical Conditions

Fasting plasma glucose data used for the supplementary diabetes analysis was from the 2015-2016 NHANES Glycohemoglobin data file.

© 2021 Herbert BM et al. *JAMA Network Open.*

Definitions

Definitions for grade of angina with associated questions and responses:

Grade 1 angina is defined as follows with ‘question’ and (response): 1) ‘Have you ever had any pain or discomfort in your chest?’ (yes), 2) ‘Do you get it when you walk uphill or hurry?’ (yes), 3) ‘What do you do if you get it while you are walking? Do you stop or slow down or continue at the same pace?’ (stop or slow down), 4) ‘If you stand still, what happens to it? Is the pain or discomfort relieved or not relieved?’ (relieved), 5) ‘How soon is the pain relieved? Would you say…’ (10 minutes or less), and 6) if the participant identifies the chest pain as in the upper or lower sternum or in the left chest and left arm. Grade 2 angina is defined as the addition of chest pain occurring when the participant walks at an ordinary pace on level ground to the grade 1 angina definition above. If any of the criteria are answered differently, the participant is categorized as not having anginal symptoms. Response options of ‘refused’ and ‘don’t know’ are possible for each question.

© 2021 Herbert BM et al. *JAMA Network Open.*
